# The Aim of the Milk Station

**Published:** 1914-12

**Authors:** F. E. Brundage

**Affiliations:** Physician to Memorial Chapel Milk Station, Buffalo. 2561 Main Street


### The Aim of The Milk Station

By F. E. BRUNDAGE, Ph. B., M. D.,

Physician to Memorial Chapel Milk Station, Buffalo.

It is surprising that with the growth of our knowledge of infant welfare and the increased public interest along this line, the real aim of the milk station is so little understood. The majority of people are not even aware that there is such an institution, not to mention their ignorance of its special purpose. And what is more to be regretted is that many physicians, either because of lack of interest or pressure of practice, are equally uninformed as to the milk station's work.

There are at present nine milk dispensaries in the city of Buffalo: seven under the supervision of the Babies Milk Dispensary and two under the direction of the board of health. Last year, which was the fourth year of their existence, the milk dispensaries reported having taken care of 939 babies or four times as many as in 1910. Of this number 515 were bottle fed, while the remaining 424 were breast fed. The medical staff of the dispensary is composed of a head physician with a physician in attendance at each station. Each is also provided with a nurse who is likewise supervised by a head nurse. The work is entirely dependent on public subscription. It costs about $1,000 a month to maintain the service, which includes nurses salaries, rent, heat, light, etc. No medicine is given free at the station; the milk, however, is supplied free to a few of the very poor, the expense of which is paid by the charity organization. To others it is supplied at cost. All creeds and nationalities take advantage of this opportunity; the majority of those who come, are Italian, Polish, Hungarian, Jewish and Colored.

The two prime factors that produce a high infant mortality are bad surroundings and bad feeding. The former nearly always implies the latter, but the converse of this is not true. No one social agency can at once destroy the tenement and the congested slum, yet the work of such societies as the milk dispensary may so help the mothers whose lives are spent in the gloom of the tenement that a knowledge of proper food and its preparation for their babe will offset partially the effect of bad surroundings. And it is on the matter of proper feeding that the milk dispensary lays its greatest, though not entire, emphasis.

In 1904 the infant mortality in Buffalo, in relation to the birth rate was 14.7% for babies under one year of age. In 1909 it was 13.7%, while so far this year it is 13%. It is therefore evident that all the forces working together for the lowering of infant mortality have accomplished a gradual decrease in the death rate. How much of the credit for this surely decreasing death rate among babies is due to the milk station cannot be determined exactly. But it is not doubtful that it is doing its full share of a work that in the aggregate is of national importance.

Our main purpose at the station is not so much to give proper care to sick babies as to maintain in health the normally well baby and to teach mothers how to secure this result. Just here the difficulty begins; for most mothers see no need of going to the milk station when a baby is well. It is comparatively easy, however, to interest them when something is wrong, for then they seek aid willingly. Ignorance, superstition and incompetence are present to a greater or less degree in most young mothers, particularly the foreign born in the crowded sections of a large city.

A brief word about the admission of a new case will make clearer the real value of the work. A Syrian mother let us say, has been told by a neighbor, nurse, or physician to bring her baby to the station for special milk. The child is perhaps unwell or she is unable to nurse it longer. She comes to the milk station where it is stripped, weighed and examined thoroughly by the physician in charge. If there exists any abnormal condition, such as phimosis, adenoids, or enlarged tonsils, the mother is referred to her family physician or a dispensary for the treatment of this particular ailment. A chart is then made out which contains every thing essential for the physician's knowledge about the child's condition. It differs from the ordinary chart and contains a number of special headings which are peculiar but necessary for this special line of work. For instance, conditions in the babe's home are thoroughly investigated by the nurse at her first visit and are then entered on the chart under such headings as these; number of rooms, light, ventilation, yard, porch, ice-box, and general cleanliness. The number and ages of other members of the family are important from the fact that here is shown whether the mother has all the work to do or whether she has some assistance from the older children. Moreover the income of the father must be known, as it supplements the information the nurse may gain at her first visit at the home. Besides the above mentioned, the ordinary facts are obtained which are found on any medical chart. After the chart is filled out, the necessary advice is given to the mother. She is told that the nurse will call the next day to teach her how to prepare the food and how to take general care of the baby in the home. She is asked to return to the milk station the next week to have the baby weighed and the weight recorded so that the progress of the child may be known. Just here it is important to say that every effort is made to win the confidence of the mother. Her impressions of the efficiency of the physician and nurse must be decided but it is equally necessary that she should feel their kindliness and friendliness. Once having realized these she is far more likely to follow the advice which is so freely given in the interest of her babe's health, for without her co-operation the station's work is entirely fruitless.

It is essential that we get the baby a few weeks old and better still if it is the first; for we know by experience that the mother of the first baby has no formed ideas and if started right will tend to follow good instruction. But the mother of several children is hard to teach, for in bringing them up she has formed opinions which are so thoroughly rooted that it is at times impossible to eradicate them. One illustration of this point is the general disregard of fixed hours for nursing. They have always fed the baby whenever it cried, and as no bad result has happened, they think it the proper way.

In directing the general care of the baby in the home, the station's nurse lays great stress on regularity in regard to feeding, bathing, sleep, proper care of the diapers, cleanliness of all utensils, careful preparation of the food and dressing the baby according to the weather rather than the calendar. Although it does not seem necessary, yet in a great many cases, we have to say that clean cows milk is the best food for the baby next to mothers milk. Some cases need special modification, such as the addition of gruels, peptonizing or different percentages of the fat or proteid. The cases are few where the baby will not digest cows milk if given a fair chance.

Almost all mothers who bring their babies to the milk station have allowed the formation of harmful habits. Among the most vicious habits we have to contend with is the pacifier abuse. It is not an exaggeration to say that fully half of the babies are victims. Even after a full discussion of the subject by the physician and nurse we are unable to eradicate this practice in some cases. The mother states that they quiet the baby when nothing else does and enables her to do a little work. She is of course unaware of the dangers of this habit: the introduction of germs into the mouth with the resulting soreness and ulceration and changes in the shape of the mouth so that it is distorted and ugly in appearance. The mother is warned of these at her first visit at the station.

Fresh air is more essential to a baby than medicine, yet it is quite difficult to convince some mothers of this fact. If the baby is born in the winter time, the mother is taught to bring the baby gradually into the fresh air. In the summer time, no matter what the age, the baby should have fresh air all the time. It is often our experience in summer to see a baby kept in a close room with no ventilation and blankets heaped upon it for fear that a draught may give the baby a cold. It is not to be wondered at that these babies develop bowel trouble in summer and diseases of the respiratory tract in the winter. Attention to the bowels in both breast and bottle fed is a matter of great importance, contrary to the ideas of some mothers. Continual constipation is not a normal condition and every effort is made to correct it. In the case of the breast fed the mother is usually at fault. She is given a diet composed mainly of vegetables and fruits and told to take plenty of exercise in the fresh air. This in most cases alleviates her condition and cures the babe of the same trouble. A bottle fed baby is more easily amenable to treatment; orange juice is given as early as three months, laxative gruels are added to the food and often a change of sugar or more of the same kind is quite sufficient. Cane sugar is used in most cases and has been found entirely satisfactory.

The feeding of solids too early is one of the greatest temptations of almost every mother. It is a common thing to find a child under a year of age fed upon such things as bananas, potatoes, cookies, and the like. It is unfortunately true that a great many babies will digest these things but they are absolutely prohibited with the milk dispensaries babies. The first thing that is advised outside of milk is orange juice which is given as a regular diet at six months of age. At eight months, beef juice or beef soup, zweibach and the white of egg, soft cooked is added. At one year of age, they are allowed stewed fruit and cooked cereals and occasionally baked potato. This is the age too, when most mothers give everything that is on the table. Every mother is given to understand that overfeeding is attended with great risk and liable to cause serious disturbance to the child. One of the troublesome times for young untrained mothers is the weaning period, and the milk station saves many a baby from the effects of a reckless guess work on the part of the mother. When properly done weaning is not attended with any bad results. It is best to start at ten months to substitute one feeding for the breast and within two months have the baby entirely weaned. Some mothers will even nurse their babies until two years of age, thinking that it is best for the baby as long as they have the milk. This kind of baby will usually show evidences of malnutrition unless some other kind of food is given. Other mothers when they become pregnant wean the baby at once believing that the milk is poisonous to the child when they could just as well take a few weeks to wean and not entirely upset the digestion. They invariably give stronger food than the baby can possibly digest. There are times, of course, when sudden weaning is imperative but it is the withdrawal at the least provocation which gives us the most trouble. For instance a mother was told that the facial eczema present on her baby was due to her bad milk and she should wean at once. She did so but gave the baby stronger milk than it could digest, consequently she brought the baby to the station with symptoms of vomiting and diarrhoea which gave a great deal of trouble for some time. We have a surprising number of these cases and they come to the dispensary for milk thinking that it will at once cure their baby.

The little mother's club has an important bearing in our work and deserves mention. It consists of girls from the ages of ten to sixteen who meet once a week at the station and learn of the nurse in charge the simple rules of caring for the baby. It is surprising at times to see the interest and enthusiasm they exhibit in their work. At their homes and at the homes of others they impart their knowledge which is indirectly the result of saving many babies. Its future use to them is also of the highest value. The next step in the work of the milk station will be directed toward prenatal care of expectant mothers. This is yet to be developed when there are sufficient funds to guarantee a permanent system. It will then be an established part of the work.

The milk station is playing a great part in the universal campaign for a lower death rate among city babies. Its location among the foreign born and the poor gives it an opportunity for good that can be accomplished in no other way. Considering the wideness of its scope and the elevating character of its purpose, it merits the support and encouragement of every citizen.

2561 Main Street.

---

**Commercialization of Human Milk.** Raymond Hubler, "Arch. of Paed.," March, 1914, states that at Bellevue 50 cents is paid each mother who has an excess of milk and who, after careful examination to exclude disease, is willing to submit to milking. The yield averages 10 ounces a milking. By this plan, the earning capacity of poor women is increased and a supply of human milk is available for cases requiring it.

---

**Relation of Time of Day to Mortality.** While we have never believed in the old notion that men went out with the ebb tide, we have always had a general impression that aside from accidental deaths, there was an excess of mortality in the last part of the night. The following statistics of Billings of London, "The Pract.," 1914, based on an analysis of 10,000 deaths in London hospitals, 1909 to 1913, show very little difference. 11 p. m. to 3 a. m., 17.06%; 3 to 7 a. m., 17.03%; 7 to 11 a. m., 15.15%; 11 a. m. to 3 p. m., 17.54%; 3 to 7 p. m., 16.83%; 7 to 11 p. m., 16.39%.

